# Evaluation of In Doped GaAs Alloys to Optimize Electronic, Thermoelectric and Mechanical Properties

**DOI:** 10.3390/ma15051781

**Published:** 2022-02-26

**Authors:** Aiyeshah Alhodaib

**Affiliations:** Department of Physics, College of Science, Qassim University, Buraydah 51452, Saudi Arabia; ahdieb@qu.edu.sa

**Keywords:** semiconductor alloys, electrical conductivity, thermal conductivity, classical transport theory

## Abstract

The electronic, mechanical and transport properties of the In substitution in GaAs are investigated by the TB-mBJ potential, BoltzTraP code and Charpin tensor matrix analysis using Wien2k code. The formation energies of the alloys Ga_1−x_In_x_As (x = 0.0, 0.25, 0.50, 0.75 and 1.0) confirm that they are thermodynamically favorable. The directional symmetry changes when increasing the In concentration and reduces the bandgap from 1.55 eV (GaAs) to 0.57 eV (InAs), as well as reducing the electrical conductivity and increasing the Seebeck coefficient. The thermoelectric performance is depicted by the power factor without including lattice vibration. The elastic properties’ analysis shows mechanical stability, and elastic moduli decrease with an increasing In in GaAs, which converts the brittle nature to ductile. The Debye temperature, hardness and thermal conductivity decrease, thus, increasing their importance for device fabrications.

## 1. Introduction

The history of human civilization is associated with the use of energy because even before our control over fire, natural food was a source of energy [1]. Although technological advancements have resulted in efficient devices that require less energy, energy demands are continuously increasing due to depleting existing energy sources and the rising human population [2,3]. Therefore, renewable energy sources have attracted huge scientific interest. The flourished microelectronic industry has made it possible to design novel and efficient devices; however, these devices also waste energy by producing heat during their operation. This, in turn, requires the useful phenomenon of thermoelectricity [4], in which heat can cause an electric current. Hence, for efficient thermoelectric performance, materials showing an optimum potential difference and charge carrier flow due to the employed thermal gradients are required to exhibit high power outputs [5]. This shows that semiconductors exhibiting narrow and direct bandgaps are potential candidates to exhibit higher thermoelectric efficiencies [6].

GaAs has a direct bandgap (1.52 eV) semiconductor [7] with a cubic zinc blende structure that is interesting for thermoelectric applications. The high electron mobility in GaAs [8] makes it appropriate for light-emitting, photovoltaic [9], high power microwave [10] and optoelectronic [11,12] devices. The alloying of GaAs with an appropriate element narrows the effective bandgap, and hence, the desired range of bandgap for the specific target applications is possible. The narrow bandgap of bulk InAs is 0.35 eV, and it stabilizes a cubic zinc blende (ZB) structure. Therefore, its alloy with GaAs over the whole compositional range can elucidate its significance for thermoelectric devices [13]. For example, embedded indium nanocrystals (NCs) have been reported to improve the number of free carriers, while increased electron and phonon scattering at boundaries suppress thermal conductivity. The carrier trapping caused a 25% increase in the Seebeck coefficient [14]. A theoretical study of the Ga_1−x_In_x_As alloys reveals that an indium (In) addition narrows the bandgap, although the magnitude of the bandgap was sensitive to the applied exchange-correlation approximation [15]. Cheap computational technology, fast processing algorithms and efficient theoretical models are available these days, suggesting that theoretical computations of the physical properties are quite reasonable. The theoretical studies of GaAs nanoclusters with impurities were performed by J. A. Rodríguez-Jiménez. The structural, electronic and magnetic properties are explored through size, while we control magnetism. In the present study, the characteristics are addressed by the substitution of In in place of Ga [16,17,18].

In this study, I employ density functional theory (DFT) based computations to investigate the impact of an indium (In) addition in GaAs (for the whole composition range of In) on the displayed thermoelectric properties. The electronic properties are determined using modified Becke–Johnson potential for getting quite accurate structures, and hence, the computed thermoelectric parameters are more consistent. The thermoelectric parameters are shown versus chemical potential and temperature, which indicate that the studied alloys are suitable for thermoelectrics.

## 2. Method of Calculations

In the current article, I have optimized the crystal structures of Ga_1−x_In_x_As (x = 0.0, 0.25, 0.50, 0.75 and 1.0) alloys by FP-LAPW, and integrated them into the Wien2k code though PBEsol approximation [19,20,21]. The FP-LAPW method treats the function in two regions: the muffin-tin region, in which the solution of the wave function is taken as a spherical harmonic and interstitial region in which the solution is considered a plane wave type. The wave function in the muffin-tin sphere is non-overlapping to avoid the electrons leakage error. The strain forces are reduced up to 0.0001 Ry by relaxing the structures before optimizing them. The convergence of the charge/energy was achieved up to 0.1 mRy through the iteration process of a self-consistent field. The k-mesh for convergence was in the order of 20 × 20 × 20 because, at this point, the energy released from the studied system becomes constant. The PBEsol approximation evaluates the ground state properties accurately but underestimates the bandgap. Therefore, the modified potential of Trans and Blaha (TB-mBJ) has been introduced to calculate the bandgap exactly because the thermoelectric behavior of the studied materials depends on the bandgap [22]. The modified Becke and Johnson potential is the most versatile, accurate, and friendly to use as compared to PBEsol and HSE06. Therefore, we implemented the TB-mBJ potential to compute the properties of studied materials. Even though the TB-mBJ and HSE06 findings are equivalent, the HSE06 is computationally expensive. The PBEsol gives the underestimated bandgaps. Furthermore, the modified structures are treated through the BoltzTraP code [23]. The thermoelectric parameters of the studied alloys have been calculated through transport coefficients, as given below:

(1) $$\sigma_{\alpha \beta }(\varepsilon )=\frac{1}{N}\sum_{i,k}\sigma_{\alpha \beta }(i,k)\frac{\delta (\varepsilon -\varepsilon_{i,k})}{\delta (\varepsilon )}$$

(2) $$\sigma_{\alpha \beta }\left(i,k\right)=e^{2}\tau_{i,k}\upsilon_{\alpha }\left(i,\vec{k}\right)\upsilon_{\beta }\left(i,\vec{k}\right)$$

where the number of matrix elements is (*N*) and group velocity is *υ_β_* (*i*, *k*). The Seebeck coefficient (*S*) and electrical conductivity (*σ*) are represented by the equations where the product gives a power factor to judge the efficiency of the thermoelectric device.

(3) $$\sigma_{\alpha \beta }(\alpha ,\mu )=\frac{1}{\Omega }\int \sigma_{\alpha \beta }(\varepsilon )[-\frac{\partial f_{0}(T,\varepsilon ,\mu )}{\partial \varepsilon }]d\varepsilon$$

(4) $$S_{\alpha \beta }(T,\mu )=\frac{1}{eT\Omega \sigma_{\alpha \beta }(T,\mu )}\int \sigma_{\alpha \beta }(\varepsilon )(\varepsilon -\mu )[-\frac{\partial f_{0}(T,\varepsilon ,\mu )}{\partial \varepsilon }]d\varepsilon$$

The electrical conductivity of the semiconductor materials is related to the bandgap and the relation to the time of the semiconductor, while the Seebeck coefficient is independent. The electrical and thermal conductivities are reported up to 1000 K because the melting temperatures of GaAs and InAs are 1513 K, and 1206 K [24,25,26]. The BoltzTraP code used to compute these conductivities is based on the classical theory of Boltzmann, which gives their behavior against temperature and chemical potential, or carrier concentrations. However, for the calculations of formation energies and phonons at 1000 K, the time-dependent density functional theory is required, which is beyond the limits of the BoltzTraP and Wien2K codes.

## 3. Results and Discussion

### 3.1. Structural and Electronic Behavior

**Figure 1:**
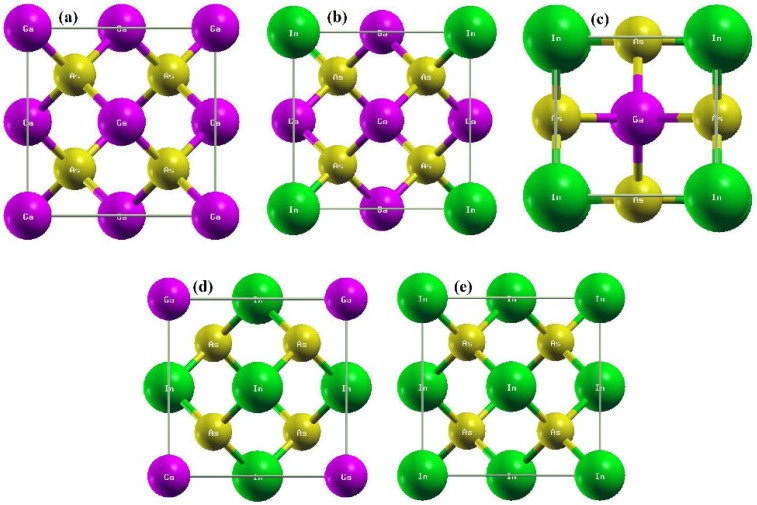
(**a**–**e**) Structures of Ga_1−x_In_x_As (**a**) x = 0.0, (**b**) x = 0.25, (**c**) 0.50, (**d**) 0.75 and (**e**) 1.0 formed by Xcrysden (blue color balls (Ga), brown color (As) and green color (In)).

The optimized structures of Ga_1−x_In_x_As (x = 0.0, 0.25, 0.50, 0.75 and 1.0) have been illustrated in Figure 1a–e. The cubic structure of GaAs with space group (216) F43m in the zinc blende (ZB) phase has been doped with In. The In doping changes the space group of binary GaAs, (216) F43m to (215) P43m (x = 0.25), (115) P-4m2 (x = 0.50), (215) P43m (x = 0.75) and (216) F43m for InAs. The changing symmetry and space groups modify the lattice parameters and bandgaps. The lattice constant increases with the increase in the doping of In in GaAs because In has a greater atomic radius than Ga, and bulk modulus decreases; the increasing atomic size makes the structures less dense due to increases in the volumetric strain. I have observed the lattice constant increase from 5.66 Å (GaAs) to 6.09 Å (InAs), while the bulk modulus decreases from 69.34 GPa (GaAs) to 57.84 GPa (InAs), as shown in Table 1. The reason for increases in the lattice constant and decreases in the bulk modulus is the small atomic size of Ga than In. Furthermore, the formation energy has been depicted to ensure the thermodynamic existence of these doping by the relation:

(5) $$\Delta H_{f}=E_{Total}\left(Ga_{l}In_{m}As_{n}\right)-lE_{Ga}-mE_{In}-nE_{As}$$

where $E_{Total}\left(Ga_{l}In_{m}As_{n}\right)$, $E_{Ga}$, $E_{In}$ and $E_{As}$ are the energies of alloys in individual elements [27]. The negative formations ensure the thermodynamic existence of these alloys. Moreover, the formation energy decreases from −0.36 eV (GaAs) to −0.23 eV (InAs) which shows GaAs is more thermodynamically stable than InAs (See Table 1). Furthermore, the positive phonon frequencies of GaAs and InAs are reported in the literature, which ensures their dynamic stability [28,29]. Therefore, the doping in between these two end binaries must be stable.

**Figure 2:**
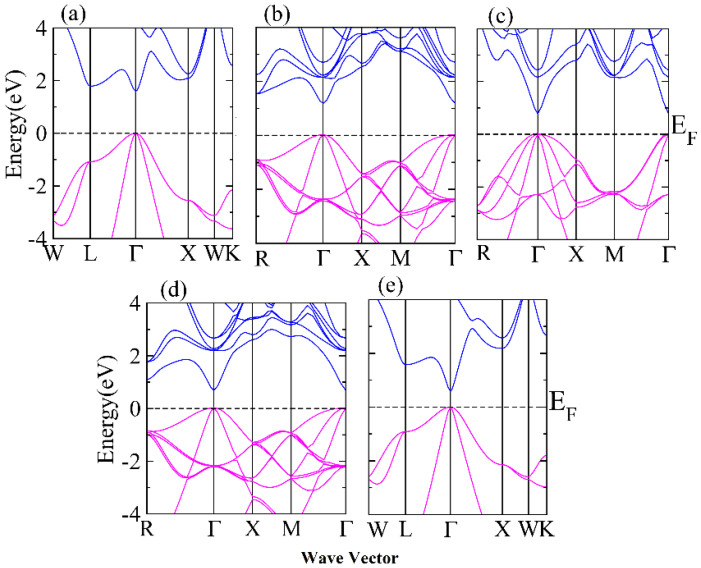
(**a**–**e**) Band structures of Ga_1−x_In_x_As (**a**) x = 0.0, (**b**) x = 0.25, (**c**) 0.50, (**d**) 0.75 and (**e**) 1.0.

The main purpose of the doping of In in GaAs is the tuning of the bandgap, which modifies the optical and transport properties of studied alloys. The valence bands and conduction bands lie at the Γ direction for all the studied compositions, which ensure the direct bandgaps. The direct bandgap decreases from 1.55 eV (GaAs) to 0.57 eV (InAs) (see Table 1). This variation of bandgap suggests the optoelectronic applications form visible to infrared devices as shown in Figure 2a–e. Moreover, the modification of the bandgap and structural symmetry changes the transport and mechanical properties, which is analyzed in brief in this article.

**Figure 3:**
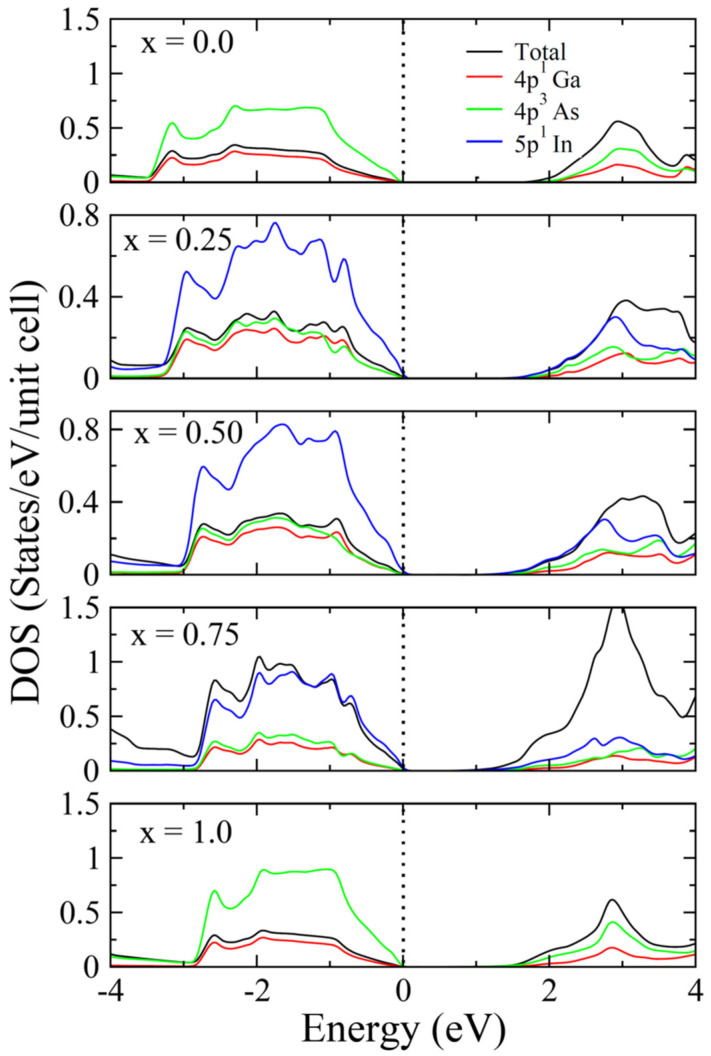
Density of states (DOS) of Ga_1−x_In_x_As (x = 0.0, 0.25, 0.50, 0.75, 1.0).

For the detailed analysis of individual contributions of elements (Ga, In, As) their DOS are plotted in Figure 3. In end binaries GaAs and InAs, the role of the 4p states of As are prominent (x = 0.0, 1.0). For the In replacement with Ga, the In 5p states have more influence than Ga. Therefore, the major transitions can take place from VB of In 5 p states to other states of Ga, As and In in CB. These transitions are important for optoelectronics and thermoelectric devices fabrications.

### 3.2. Transport Properties

**Figure 4:**
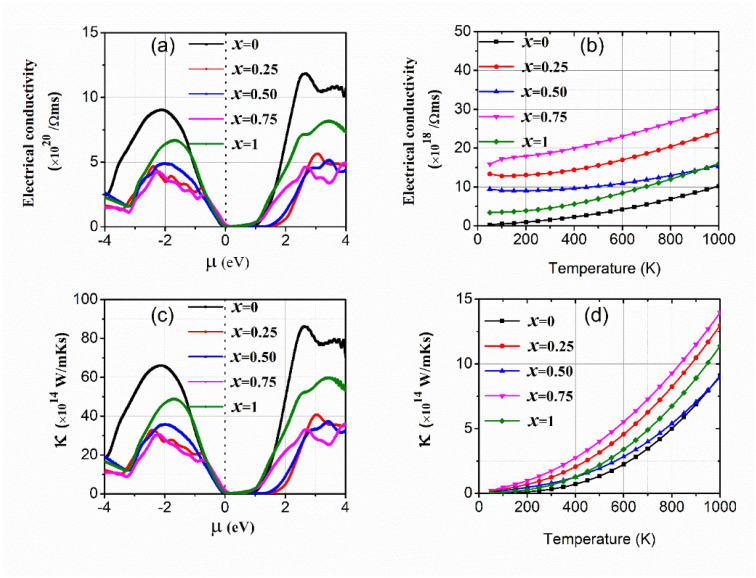
(**a**,**b**) Electrical conductivity *σ* and (**c**,**d**) thermal conductivity *κ* of Ga_1−x_In_x_As against chemical potential and temperature.

The deficiency of energy sources and more needs in the world inspired scientists to work on new avenues to produce energy. It is necessary to study thermoelectric materials for the conversion of heat into useful electrical energy [30,31]. The materials with a narrow bandgap improve the device’s thermal efficiency [32,33,34]. The electrical conductivity (*σ*) delivers a route to the carriers for the conduction process in semiconductors affected by the bandgap. Here, τ is the relaxation time which is fixed in the BoltzTraP code for the value 10^−14^ s [26]. The reported values of *σ* against chemical potential (*µ*) and kelvin temperature (T) are presented in Figure 4a,b. The μ is the measure of energy, important for the elimination or the incorporation of electrons against coulomb repulsive forces. The +Ve and −Ve values of *µ* distinguish the *n*-type and *p*-type regions. The concentration of holes increases up to 8.5 × 10^20^/Ωms (2.1 eV), 7 × 10^20^/Ωms (1.8 eV) and 5 × 10^20^/Ωms (2.2 eV) for GaAs, InAs, and doped alloys, respectively. Similarly, in the *n*-type region it reaches peaks of 13 × 10^20^/Ωms (2.5 eV), 8 × 10^20^/Ωms (3.0 eV), and 5.2 × 10^20^/Ωms (3.2 eV) for GaAs, InAs, and doped alloys, respectively. Therefore, binaries have more holes and electrons. The comparison also shows more electrons are accessible in the *n*-type region than holes in the *p*-type region. The trends of electrical conductivity versus temperature are in Figure 4b, in the temperature range from zero to 1000 K. It is found that the *σ* increases with increasing temperature because increasing temperature requires more carriers for conductions. Furthermore, the doping of In in GaAs also has a significant effect on the *σ*. The *σ* has a large value at x = 0.75 and the least value for GaAs. However, the symmetry variation and changing space group also change the *σ*. The *σ* decreases in the order of x = 0.75>, x = 0.25>, x = 0.50>, x = 1.0>, x = 0.0 according to the variation of space group. At high temperatures, the carriers of In doping (x = 0.5 and 1.0) become equal, due to which the curves meet each other. The other reason is that at x = 0.50, the coulomb forces become prominent, which reduces the electrical conductivity at x = 0.50 and it becomes similar at x = 1.0.

The thermal conductivity (*κ*) of spinel materials is described by the flow of carriers. The *κ* depends upon the contribution of electrons (k_e_) and phonons (k_p_) [35]. The phonons’ contribution in wide bandgap materials is slightly small because the involvement of electrons is more prominent in semiconductor materials. In wide bandgap semiconductors, the inter-band transitions are more dominated by the intra-band transitions that eradicate the phonons’ contribution. Moreover, the phonon calculations are very costly and out of the scope of classical transport theory-based BoltzTraP code. Therefore, I only discussed the electronic part of *κ*, which is depicted in Figure 4c,d, against chemical potential and temperature. The patterns of *κ* are like *σ* but its value is ultra-low compared to the *σ*. The ratio of *κ*/*σ* can be functional in describing the semiconductor materials useful for thermoelectric applications [36,37]. Its ratio is 10^−5^, which is ridiculously small, which instigates them promising materials for thermoelectric applications.

**Figure 5:**
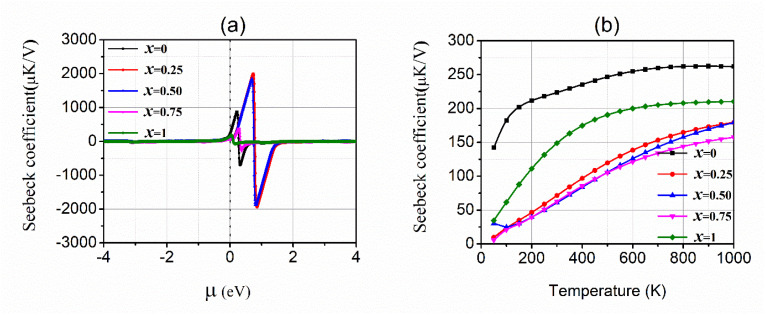
The Seebeck coefficients S of Ga_1−x_In_x_As (x = 0.0, 0.25, 0.50, 0.75, and 1.0) against (**a**) chemical potential and (**b**) temperature.

**Figure 6:**
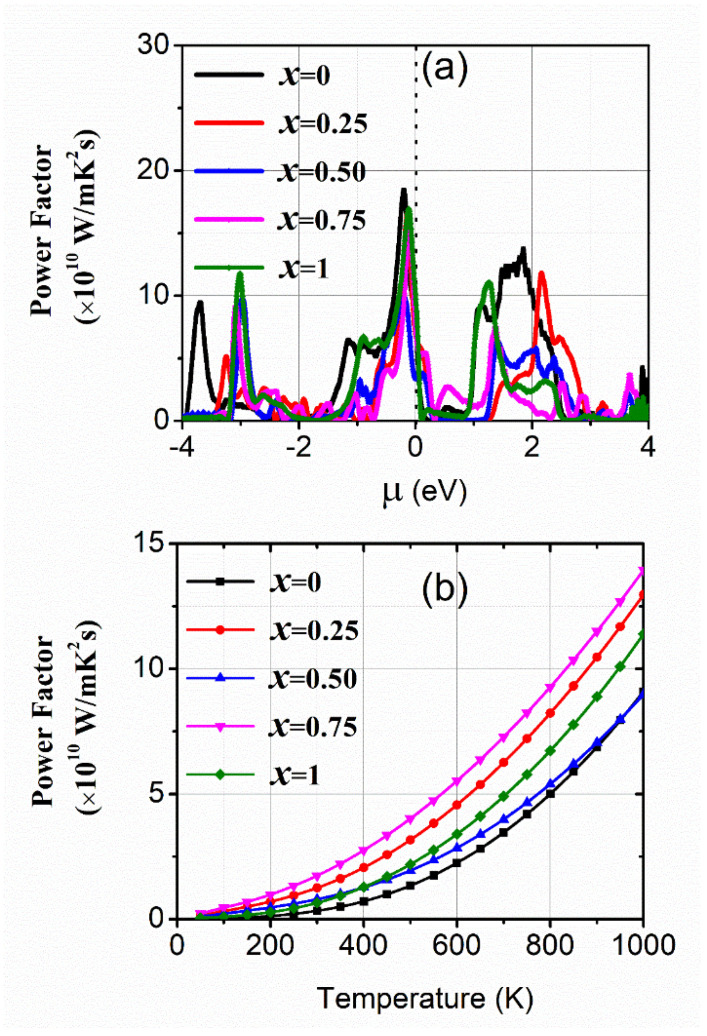
The power factors (σS^2^) of Ga_1−x_In_x_As (x = 0.0, 0.25, 0.50, 0.75, and 1.0) against (**a**) chemical potential and (**b**) temperature.

The computed values of Seebeck coefficients (S) are depicted in Figure 5a,b. This demonstrates a negative or positive value depending on most electrons or holes, respectively. The analyzed results are represented in Figure 5a, which demonstrates that the values of the Seebeck coefficient are zero in the *p*-type region. The electrons have a major character in the *n*-type region, as defined above. Its highest value changes from 2000 µV/K to −2000 µV/K for 25% and 50% concentrations of In, and the minimum value varies from 400 µV/K to −400 µV/K for x = 0.0, 75% and 100% concentration of In (*p*-type region). The Seebeck coefficient versus temperature has been depicted in Figure 5b, which shows the GaAs and InAs have a large value of Seebeck coefficient while the doped alloys have less Seebeck coefficient. Therefore, doping increases the conductivity. The execution of studied alloys has been explained in terms of the power factor which depends upon *σ* and the square of S. The reported values of PF are shown in Figure 6a,b against *µ* and T. The computed power factor for all concentrations of In exhibit more significant peaks in the *p*-type region than in the *n*-type region near the Fermi level; this means the holes’ contribution is more efficient. The PF increases with T increases, due to more values of *σ* (Figure 6b). The plots demonstrating the peak at 75% concentration show the highest value of the PF and the remaining concentrations follow the trend of *σ*. Therefore, it is analyzed that the high symmetry concentration (x = 0.75) is most suitable for thermoelectric efficiency.

It can be seen from Table 2, the highest power factor for x = 0.75 is the consequence of a high *σ* and a low value of S.

### 3.3. Mechanical Properties

The elastic behavior of studied materials has been elaborated by three coefficients of tensor matrix coefficients *C*_11_, *C*_12_ and *C*_44_ [38]. The Born stability criteria ($C_{11}$ − $C_{12}$) > 0, $C_{11}$ > 0, $C_{44}$ > 0 and *C*_11_ > *B* > *C*_12_ [39] are confirmed. The elastic constants decline with increasing In in GaAs. All elastic parameters are depicted from formulas in Ref [40]. The moduli are decreases with increasing doping concentrations, which makes the materials less dense. The plasticity of the material is explicated by Poisson’s ratio v whose condition is 0<v<0.5, which shows high plasticity of the studied materials. The v and B/G explained distinguish between a brittle and ductile nature, whose limits are B/G > 1.75 and v > 0.25 for ductile [41]. Therefore, according to these conditions, the studied material has a brittle nature at x = 0, 0.25 and with a 0.50 concentration of In, and a ductile nature at x = 0.75 for pure GaAs (See Table 3). The doping In in GaAs changes the brittle nature of alloys. The anisotropy factor (A) reveals isotropic and anisotropic behavior. For isotropic behavior, its value is 1 and a deviation from 1 (A ≠ 1) shows anisotropic behavior. Table 3 shows the studied alloys are anisotropic. The anisotropic value increases with the increase in doping of In in GaAs. The Kinmen parameter (ξ) illustrates the bond bending and bond stretching; its values toward zero represent bonding, and toward unity, it represents bond stretching. At low doping of In, the bond bending and bond stretching are similar, but at higher doping, the bond stretching is dominant over bond bending, as depicted in Table 3.

To determine the thermo-dynamical properties of the cubic crystal structure, the Debye temperature (*θ_D_*), thermal conductivity (*κ*_min_), and melting temperatures (T_m_) are reported in Table 3. Additionally, the melting temperature must be calculated [42,43]. The Debye temperature (*θ_D_*) has been calculated using the Navier equation of states in terms of average sound velocity (*v_m_*) which is expressed by the formula:

$$\theta_{D}=\frac{h}{\kappa_{B}}{\left[\frac{3n}{4\pi }\frac{N_{A}\rho }{M}\right]}^{\frac{1}{3}}\upsilon_{m}$$

where $\theta_{D}$ is Debye temperature and *N_A_*, is Avogadro number. The value of $\theta_{D}$ decreases with an increasing doping concentration of In in GaAs up to x = 0.75. It means the doping depressed the value of $\theta_{D}$ because of the depression of atomic oscillations. The variation in Debye temperature directly affects the specific heat capacity that is proportional to ${\left(T/\theta_{D}\right)}^{3}$ [44,45]. The melting point reported from mechanical parameters decreases with increasing the In concentration. The resistance of the compound under extreme conditions may also be attributed to its hardness, known as Vickers hardness.

H_V_ = 0.92 (G/B)^1.137^ G^0.708^

This hardness factor expresses the material’s capacity to resist being dented. Its value declines doping of In, as shown in Table 3, which reduces the resistance ability of the materials.

## 4. Conclusions

In short, I have evaluated the electronic, mechanical and transport properties of Ga_1−x_In_x_As (x = 0.0, 0.25, 0.50, 0.75 and 1.0) alloys. The formation energy calculations ensure that the studied alloys are stable and favorable for device preparation. The bandgap changes from 1.55 eV to 0.57 with an optimal value of 1.12 eV at x = 0.25 for optoelectronic applications. Additionally, it was found that electrical conductivity declines and the Seebeck coefficient raise with In doping in GaAs. The higher power factor is reported at x = 0.75 and x = 0.25. The Born mechanical criteria show their mechanical stability. The In incorporation converts the brittle nature to ductile. The minimum value of thermal conductivity decreases with an increasing In concentration in GaAs. Therefore, by tuning the bandgap, ductile behavior, the large power factor and ultra-low value of thermal conductivity increase the suitability for devices.

## Figures and Tables

**Table 1:** *a_o_* (Å): Lattice constant; B_o_ (GPa): Bulk modulus; *H_f_* (eV): Formation energy; and E_g_ (eV): Bandgap, for Ga_1−x_In_x_As (x = 0.0, 0.25, 0.50, 0.75 and 1.0).

Parameter	x = 0.0	x = 0.25	x = 0.50	x = 0.75	x = 1.0
*a_o_*	5.66	5.75	5.90	5.97	6.09
B_o_	69.34	66.12	63.56	60.16	57.84
*H_f_*	−0.36	−0.32	−0.27	−0.25	−0.23
E_g_	1.55	1.25	0.90	0.73	0.57

**Table 2:** The calculated transport parameters at 300 K of Ga_1−x_In_x_As (x = 0.0, 0.25, 0.50, 0.75, and 1.0) over Fermi level.

Composition	σ (×10^18^/Ωms)	κ (×10^13^ W/mKs)	Seebeck Coefficient (μV/K)	PF (×10^10^ W/mK^2^s)
GaAs	1.57	3.32	223.63	7.30
Ga_0.75_In_0.25_As	13.58	12.48	71.47	6.94
Ga_0.50_In_0.50_As	9.29	7.90	60.78	3.42
Ga_0.25_In_0.75_As	18.80	17.36	62.70	8.39
InAs	4.58	6.50	148.83	7.50

**Table 3:** The computed *C*_11_, *C*_12_ & *C*_44_, moduli (B, G, E), Pugh’s ratio (B/G), Poisson ratio (υ), anisotropy factor (A), Kinmen parameter (ξ), sound velocity Vm (Km/sec), Debye temperature *θ_D_* (K), melting temperature Tm (K), hardness Ha (GPa) and thermal conductivity *K*_min_ (Wm^−1^K^−1^) of Ga_1−x_In_x_As (x = 0.00, 0.25, 0.50, 0.75, 1.00).

Parameters	x = 0.0	x = 0.25	x = 0.50	x = 0.75	x = 1.0
*C*_11_ (GPa)	99	91	83	77	71
*C*_12_ (GPa)	43	42	41	39	38
*C*_44_ (GPa)	51	46	41	38	34
B (GPa)	61	58	55	52	49
G (GPa)	41	36	31	26	25
E (GPa)	99	88	79	73	65
B/G	1.53	1.63	1.75	1.80	1.92
*υ*	0.23	0.24	0.25	0.26	0.28
A	1.82	1.90	1.95	2.0	2.06
ξ	0.56	0.58	0.61	0.63	0.65
*V_m_* (Km/s)	5.06	5.85	5.66	5.39	3.74
*ϴ_D_* (K)	321	295	268	253	312
Tm (K)	1138	1090	1043	1008	972
H_a_ (GPa)	184	148	115	101	80.2
*K*_min_ (Wm^−1^K^−1^)	0.28	0.26	0.25	0.23	0.21

## Data Availability

The data of this paper will be available on demand.
